# Efficacy and Safety of Surgical Ligation versus Endovascular Embolization for Type II Congenital Extrahepatic Portosystemic Shunt

**DOI:** 10.1155/2021/9951393

**Published:** 2021-05-31

**Authors:** Jinlong Zhang, Weidong Duan, Zhuting Fang, Maoqiang Wang, Li Cui, Yanhua Bai, Xiaohui Li, Qicong Du, Mengqiu Shen, Feng Duan

**Affiliations:** ^1^Institution of Oncology, Chinese PLA General Hospital, Beijing 100853, China; ^2^Department of Hepatobiliary Surgery, First Medical Center of Chinese PLA General Hospital, Beijing 100853, China; ^3^Shengli Clinical Medical College of Fujian Medical University, Fuzhou 350122, China; ^4^Department of Interventional Radiology, Fujian Provincial Hospital, Fuzhou 350001, China; ^5^Department of Radiology, First Medical Center of Chinese PLA General Hospital, Beijing 100853, China

## Abstract

**Objective:**

To evaluate the safety and efficacy of surgical ligation and endovascular embolization for the treatment of type II congenital extrahepatic portosystemic shunt **(**CEPS).

**Methods:**

In this retrospective study, 23 patients diagnosed with type II CEPS between March 2011 and April 2019 were divided into either a surgical group (*n* = 13; 41.5 ± 19.9 years) or the interventional group (*n* = 10; 44.9 ± 19.7 years). The surgical group underwent laparoscopic surgical ligation of the shunt alone or ligation of the shunt and splenic artery and/or vein. The interventional group underwent endovascular embolization using microcoils, detachable coils, and vascular plug.

**Results:**

All 23 patients received a one-step shunt closure, and their clinical symptoms were significantly improved within 3-month postprocedure and without recurrence during follow-up. The serum ammonia levels in both groups decreased after the procedure and dropped to normal level at 6- to 12-month postprocedure. Compared with baseline, the portal vein diameter in interventional group increased significantly at 3-, 6-, 12-, and 36-month postocclusion (*P* = 0.01 for all). The procedure time was shorter in the interventional group (127.0 ± 43.2 minutes) than the surgical group (219.8 ± 56.7 minutes; *P* < 0.001). The intraoperative blood loss in the interventional group (32.0 ± 62.5 mL) was less than that in the surgical group (238.5 ± 396.9 mL; *P* = 0.001).

**Conclusion:**

Both surgical ligation and endovascular embolization are effective in the treatment of type II CEPS. Endovascular embolization has the advantages of shorter procedure time and less intraoperative blood loss. The ligation of the portosystemic shunt and splenic artery and vein is feasible with apparent safety, and it could avoid a second surgical treatment.

## 1. Introduction

A congenital extrahepatic portosystemic shunt (CEPS), also known as an Abernethy malformation, is classified based on the extent of intact hypoplastic portal veins. Type I CEPS is characterized by an end-to-side portocaval shunt and the absence of intrahepatic portal vein branches, while type II CEPS has some hypoplastic intrahepatic portal veins preserved with a side-to-side portocaval shunt diverting the portal vein blood to the inferior vena cava (IVC) [1–3]. Although CEPS is a rare clinical disease with less than 400 cases reported, it may develop severe complications, such as hepatic encephalopathy, pulmonary arterial hypertension, hepatopulmonary syndrome, or nodular liver lesions, among others [4].

To date, most of the published articles regarding CEPS are case reports, with a few small-sample clinical studies. Therefore, no consensus is achieved in the treatment of CEPS [4]. The recommended strategies for asymptomatic patients with CEPS are keeping them under careful observation or prophylactic interventional therapy [4]. Medical treatment is primarily used to improve symptoms caused by CEPS or its complications. While liver transplantation is the only curative treatment for type I CEPS, shunt occlusion by surgical ligation or endovascular embolization is the most common treatment for type II CEPS. The use of shunt occlusion depends on the expertise of local surgeons and the length and caliber of the shunts; however, a comparison of the two occlusion modalities in the management of type II CEPS has not been reported. We therefore retrospectively compared the safety and efficacy of surgical ligation and endovascular embolization in the treatment of type II CEPS from two medical centers.

## 2. Materials and Methods

### 2.1. Study Design and Patient Population

This was a retrospective clinical study performed at two medical centers in accordance with the Declaration of Helsinki and approved by the respective institutional review boards. All patients provided written informed consent prior to treatment. All patients in this study have not been reported.

This retrospective study included consecutive patients diagnosed with CEPS through abdominal ultrasound, computed tomographic angiography (CTA), and/or magnetic resonance imaging (MRI) between March 2011 and April 2019 in two hospitals. Study patient selection was performed in conjunction with radiologists, hepatobiliary surgeons, and interventional radiologists. Patients diagnosed with type II CEPS who presented with one malformation and at least one apparent CEPS manifestation or complication, including hepatic encephalopathy, gastrointestinal bleeding, pulmonary hypertension, hepatic myelopathy, hepatopulmonary syndrome, hepatic cirrhosis, or hepatic adenoma, were included in the study. Patients with type I CEPS, asymptomatic patients, and patients with multiple malformations, hepatocellular carcinoma, coagulopathies, active infection, chronic renal failure, or contraindications to angiography and surgery were excluded.

Initially, portal venography by way of systemic venous access and traversal of the portosystemic shunt and a 15 min shunt balloon occlusion test were performed. Patients with a large and short shunt, which may have a high risk of embolic material migration or portal venous pressure (PVP) > 25 mmHg after occlusion, underwent surgical ligation (surgical group), with all others undergoing endovascular embolization (interventional group).

### 2.2. Portography and Shunt Balloon Occlusion Test

To evaluate hemodynamics, measure PVP, and assess whether the shunt vessel could be closed, portal venography and shunt balloon occlusion test were conducted.

In general, the right femoral artery and femoral vein were cannulated using the Seldinger technique under local anesthesia or a transjugular or percutaneous transhepatic approach if the transfemoral vein approach failed. An indirect portal venography was then performed via selective angiography of the celiac artery, splenic artery, and superior mesenteric artery. Next, direct venography of the inferior mesenteric vein, splenic vein, and portal vein was performed. Lastly, a balloon occlusion test was performed (Fogarty, Thru Embolectomy Catheter, Edwards Lifesciences LLC, Irvine, CA, USA; Boston Scientific, Watertown, Mass) in the infrahepatic segment of IVC or shunt to detect the intrahepatic portal branches and measure PVP before and 15 min after balloon occlusion. If the shunt was suitable for endovascular embolization and endovascular occlusion, endovascular embolization and endovascular occlusion of the shunt were performed at the same time.

### 2.3. Laparoscopic Surgical Ligation

Under general anesthesia, a catheter was placed in the portal vein via the shunt through the femoral vein to monitor PVP. A 12 mm port for optics was placed in the umbilical area, and three 5 mm working ports were placed in the upper abdomen, right lower abdomen, and right lateral abdomen, respectively.

If PVP was <25 mmHg after 15 min of occlusion and without signs of intestinal wall edema and redness, the shunt was ligated alone. If PVP was >25 mmHg and/or there were signs of intestinal wall edema and redness, the splenic artery or splenic artery combined with splenic vein was dissected and ligated; if PVP was <25 mmHg without signs of intestinal wall edema and redness after 15 min, the splenic artery alone or splenic artery combined with splenic vein was taped. Otherwise, partial occlusion of the shunt was performed and the shunt was completely occluded during the follow-up which was called “two-step closure.”

### 2.4. Endovascular Shunt Occlusion

The selection of the embolic materials were dependent on the length and diameter of the shunt vessels. The embolization materials used were either one or a combination of 0.018-inch pushable microcoils (MicroNester; Cook Medical, Bloomington, USA), a 0.018-inch detachable coil (Interlock; Boston Scientific, Marlborough, Massachusetts, USA), and an Amplatzer vascular plug (16 mm, 18 mm, 20 mm, or 22 mm AVP IIA; Abbott Park, Illinois，USA). After selective shunt catheterization, the microcoils and interlock coil were inserted through a coaxial 2.7 F microcatheter (Progreat; Terumo, Tokyo, Japan); the plug was inserted through a 6 to 10 F long sheath (Cook medical, USA). Indirect portography through SMA was performed to detect whether there were residual shunts.

### 2.5. Postprocedural Treatment

To prevent portal and mesenteric thrombosis, 30 mg of subcutaneous enoxaparin was administered every 12 h, beginning 12 h after the procedure and throughout 7 days of in-hospital observation. Patients were then discharged if no complications occurred, and 10 mg of oral rivaroxaban was administered once daily for 30 days.

Abdominal ultrasound and contrast-enhanced CT performed at 1 week and 1 month after the procedure determined whether portal vein system thrombosis had occurred. If there was an absence of thrombosis 1 month after the procedure, rivaroxaban was stopped.

### 2.6. Follow-Up and Clinical Assessment

Follow-up at 3, 6, and 12 months after the procedure and every 12 months thereafter included clinical status, routine blood tests, serum ammonia, liver function, coagulation, and abdominal contrast-enhanced CT. The diameter of the main portal vein was measured by two radiologists independently, with the mean value used as the final result.

The primary outcomes were defined as the improvement of the clinical symptoms, including hepatic encephalopathy, gastrointestinal bleeding, hepatic myelopathy, hemoptysis, and fatigue. The secondary outcomes were defined as the increase of portal vein diameter and the improvement of hemoglobin, serum ammonia, and Child-Pugh scores.

### 2.7. Statistical Analysis

Continuous variables were recorded as means ± standard deviations, and categorical data as numbers and percentages. All data were tested for normality using a Shapiro-Wilk test. Differences in PVP, hemoglobin, serum ammonia, portal vein diameter, and Child-Pugh score pre- and postprocedure within each group were compared with paired Student's *t*-test (normally distributed) or paired Wilcoxon rank-sum test (not normally distributed). Differences between groups were tested using a Student *t*-test or Wilcoxon rank-sum test. Differences in the main symptoms, comorbidities, and location of shunt vessels between groups were tested using the two-tailed Fisher exact test. *P* < 0.05 indicated a statistically significant difference (SPSS Statistics for Windows, version 20.0; IBM, Armonk, NY).

## 3. Results

### 3.1. Patient Characteristics

**Figure 1:**
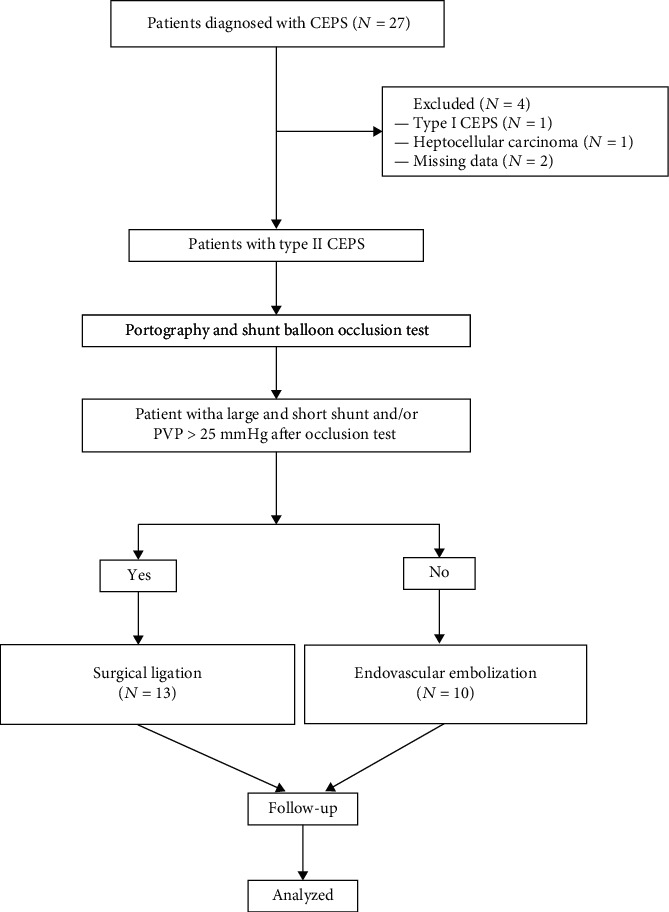
The flowchart shows the study population and groups. CEPS: congenital extrahepatic portosystemic shunt; PVP: portal venous pressure.

Baseline demographic and clinical data of the study patients are presented in Table 1. A total of 27 patients were recruited; four cases were excluded due to type I CEPS (*n* = 1), hepatocellular carcinoma (*n* = 1), and incomplete data (*n* = 2). Twenty-three patients were included in the final analysis, 13 patients (five male, eight female; mean age, 40.7 years ± 20.4; age range, 3-67 years) in the surgical group and 10 patients (eight males, two females; mean age, 44.9 years ± 19.7; age range, 17-69 years) in the interventional group (Figure 1).

The main manifestations of CEPS in this cohort were hepatic encephalopathy (six in the surgical group and five in the interventional group) and gastrointestinal bleeding (four in the surgical group and two in the interventional group) with no significant difference between the groups (*P* > 0.99 and *P* = 0.66, respectively). Other manifestations included dyspnea, abdominal pain, hepatic myelopathy, hemoptysis, and fatigue, with no significant differences between groups (*P* > 0.99 for all).

Of the 13 patients in the surgical group, 10 experienced hepatic cirrhosis, two experienced hepatic adenoma, one experienced pulmonary hypertension, and one experienced hypersplenism. Similarly, of the 10 patients in the interventional group, five experienced hepatic cirrhosis, one experienced pulmonary hypertension, and one experienced hypersplenism.

### 3.2. Location of Shunt Vessels

**Figure 2:**
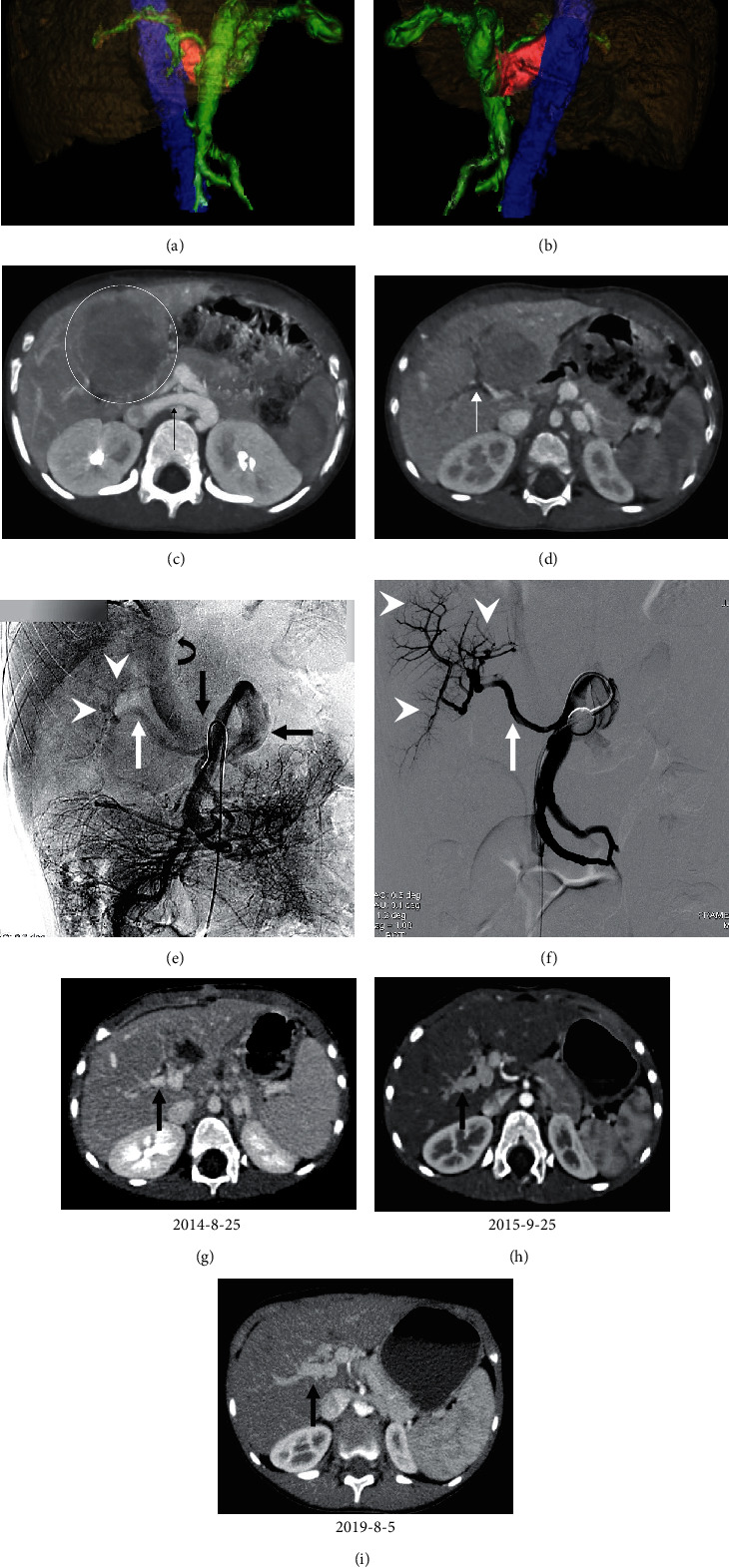
Images in a 3-year-old girl diagnosed with type II CEPS with hepatic encephalopathy (HE) who underwent surgical ligation due to large portal vein-IVC shunt. (a) Virtual reality (VR) images reconstructed from computed tomographic angiography (CTA) data prior to ligation. Anteroposterior view shows the portal vein and its branches (green), inferior vena cava (IVC) (blue), and the patent shunt communicating portal vein and IVC (red). (b) Posteroanterior VR image shows the portal vein and its branches (green), IVC (blue), and the portocaval shunt (red). (c) Axial CT image shows the large portal vein-IVC shunt (black arrow) and hepatic adenoma (circle) diagnosed by biopsy. (d) Axial contrast-enhanced CT image shows hypoplastic intrahepatic portal veins (white arrow) preoperative. (e) Indirect portal venography via the superior mesenteric artery (SMA) demonstrates venous outflow of the superior mesenteric vein (SMV) through the shunt (black arrow) drained into IVC (curved arrow), main portal vein (white arrow), and hypoplastic intrahepatic portal vein branches (arrowhead) which are visible. (f) Portal venography with balloon occlusion shows fine main portal vein (white arrow) and hypoplastic intrahepatic portal vein branches (arrowhead). The portal venous pressure (PVP) is 14.7 and 16.5 mmHg before and 15 min after balloon occlusion, respectively. (g) Contrast-enhanced CT images demonstrate the intrahepatic portal veins at 1 month after surgical ligation. (h) CT scan shows the intrahepatic portal veins at 12 months after surgical ligation. (i) CT scan shows the intrahepatic portal veins at 60 months after surgical ligation; the portal vein grows well over time.

**Figure 3:**
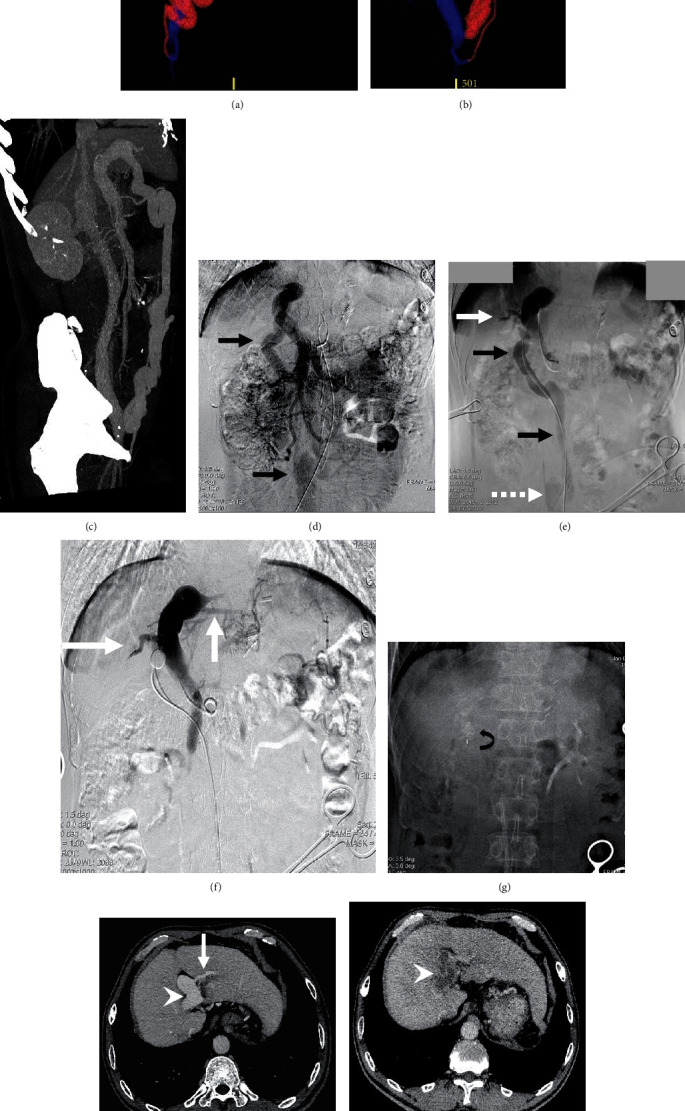
Images in a 54-year-old male diagnosed with type II CEPS with HE who underwent endovascular embolization. (a) Anteroposterior VR image reconstructed from CTA data prior to occlusion shows portal vein and its fine intrahepatic branches (green), IVC (blue), and tortuous and dilated portal vein-iliac vein shunt via the paraumbilical vein (red). (b) Sagittal VR image shows portal vein and its branches (green) IVC (blue) and the shunt (red). (c) Sagittal maximum intensity projection at the portal venous phase demonstrates the portal vein, IVC, and the shunt, in accordance with the VR findings. (d) Indirect portal venography via SMA demonstrates portal venous outflow drained into a tortuous and dilated shunt (black arrow). (e) Portal venography demonstrates partial hypoplastic intrahepatic portal venous veins (white arrow) and the communication between the portal vein and right iliac vein (dotted arrow) via the shunt (black arrow). (f) Portal venography with balloon occlusion shows hypoplastic intrahepatic portal veins (white arrow). The PVP is 21.7 and 24.3 mmHg before and 15 min after balloon occlusion, respectively. (g) The shunt was embolized with Amplatzer plug (curved arrow). (h) Preoperative contrast-enhanced CT image demonstrates the dilated main portal vein (arrowhead) and the hypoplastic intrahepatic portal vein branches (white arrow). (i) CT scan shows portal vein thrombosis (arrowhead) at 1 month after the procedure which disappeared after seven-day anticoagulation therapy. (j) Contrast-enhanced CT images demonstrate the main portal vein shrinking into normal level (arrowhead), and the intrahepatic portal veins (white arrow) grow well at 12 months after interventional occlusion. (k) CT scan demonstrates that the intrahepatic portal veins (white arrow) grow well at 36 months after the procedure.

In the surgical group, the types of shunt observed were splenorenal shunt (*n* = 5), superior mesenteric vein-inferior vena cava shunt (SMV-IVC; *n* = 3), portal vein-IVC shunt (*n* = 3), SMV-renal vein shunt (*n* = 1), and portal vein-renal vein shunt (*n* = 1), while in the interventional group, splenorenal shunt (*n* = 3), SMV-IVC (*n* = 1), portal vein-IVC shunt (*n* = 2), SMV-renal vein shunt (*n* = 1), portal vein-renal vein shunt (*n* = 1), and portal vein-iliac vein shunt (*n* = 2) were observed (Table 1; Figures 2 and 3).

### 3.3. Intraoperative Parameters

All 23 patients received a one-step closure. For patients in the surgical group, three underwent shunt ligation alone, six underwent shunt and splenic artery ligation, and four patients underwent ligation of the shunt and splenic artery and vein. The postprocedure PVP (17.5 ± 3.8 mmHg; range: 8.1-22.1 mmHg) was significantly greater than the preprocedure PVP (12.9 ± 3.7 mmHg; range: 5.9-17.6 mmHg; *P* < 0.001) in the surgical group, but it was lower than 25 mmHg. In the interventional group, the shunts were embolized using an Amplatzer vascular plug in four patients, with the remaining six cases using a combination of detachable and pushable coils (Figures 2 and 3). The PVP were not significantly different pre- and postocclusion (pre: 15.9 ± 4.7 mmHg; range: 11.4-21.7 mmHg; post: 17.1 ± 4.8 mmHg; range: 13.2-24.3 mmHg; *P* = 0.22) (Table 2).

The procedure time was significantly shorter in the interventional group (127.0 ± 43.2 min) than that in the surgical group (219.8 ± 56.7 min; *P* < 0.001). The intraoperative blood loss in the interventional group (32.0 ± 62.5 mL) was significantly less than that of the surgical group (238.5 ± 396.9 mL; *P* = 0.001). Additionally, the treatment expense of the interventional group (46331.5 ± 18839.1 yuan) was lower than that of the surgical group (59561.3 ± 21450.1 yuan) but without statistical significance (*P* = 0.20) (Table 3).

### 3.4. Clinical Outcomes

The mean follow-up period was 26.1 ± 17.6 months (range, 6-60 months). Clinical symptoms of all patients significantly improved within 3 months of their procedure without recurrence during follow-up. Serum ammonia levels decreased after both procedures to normal levels (9-30 *μ*mol/L) at 6- to 12-month postprocedure. The symptoms of hepatic encephalopathy and hepatic myelopathy associated with hyperammonemia were improved within 1 month of the procedure and remained improved during the follow-up period (Table 4).

Patients with gastrointestinal bleeding (four in the surgical group and two in the interventional group) stopped bleeding after their procedure. One patient in the surgical group suffered hematemesis 6 months after occlusion. Gastroscopy revealed hemorrhage of a gastric ulcer, and bleeding stopped after endoscopic and acid suppression therapy. One patient with hemoptysis and one with dyspnea related to pulmonary hypertension achieved symptomatic relief at 1 week and 3 weeks after the procedure, respectively. Finally, one patient with hepatic adenoma underwent simultaneous resection.

**Figure 4:**
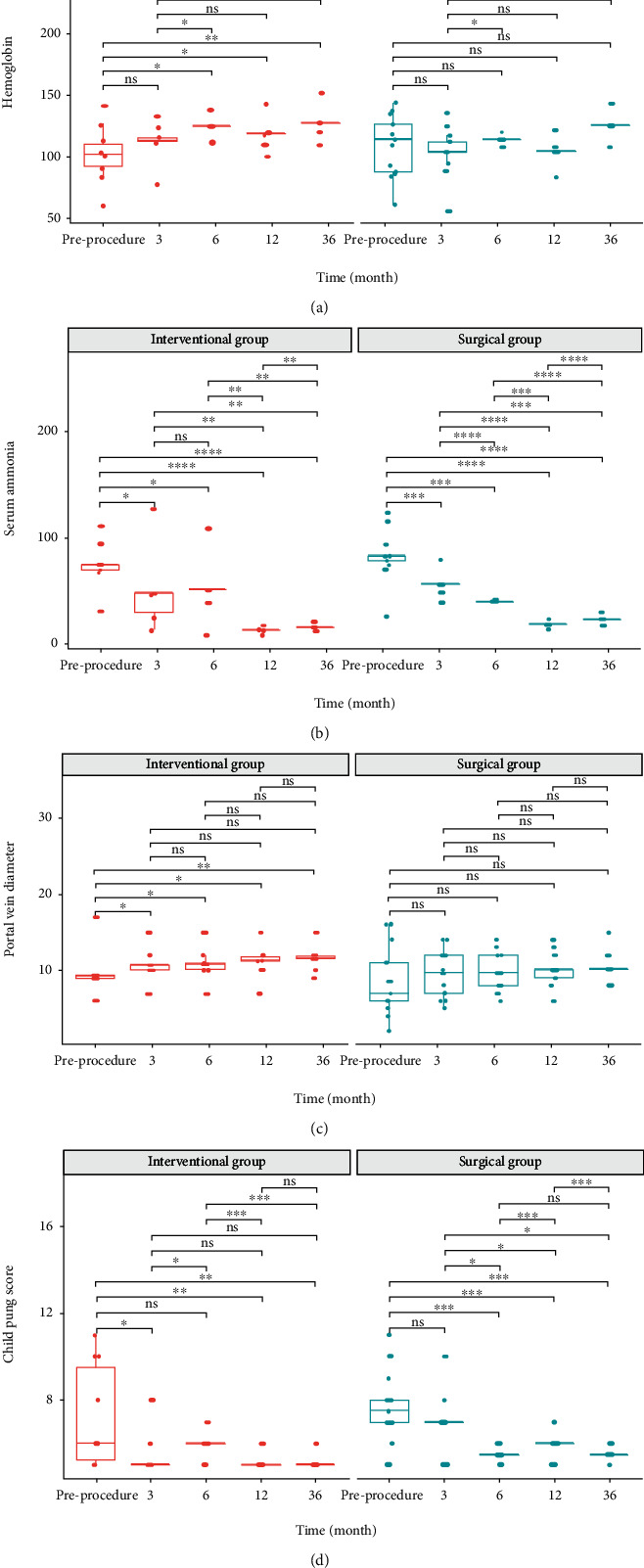
(a) Within-group comparison of hemoglobin in the interventional and surgical group pre- and postprocedure. (b) Within-group comparison of serum ammonia in the interventional and surgical group pre- and postprocedure. (c) Within-group comparison of portal vein pressure in the interventional and surgical group pre- and postprocedure. (d) Within-group comparison of Child-Pugh score in the interventional and surgical group pre- and postprocedure. Note: 0 “^∗∗∗^”; 0.001 “^∗∗^”; 0.01 “^∗^”; 0.05 “.”; 0.1 “” 1.

Compared with baseline, the portal vein diameter in the interventional group increased significantly at 3-, 6-, 12-, and 36-month postocclusion (*P* = 0.01 for all), but there was no significant difference in the surgical group (*P* > 0.05 for all) (Table 4; Figure 4).

Portal vein thrombosis was detected in one patient in the interventional group using contrast-enhanced CT scan at 1 month after the procedure. Rivaroxaban was discontinued; intravenous heparin sodium (40 U/kg to control the activated partial thromboplastin time between 40 and 60 s) and urokinase (4000 U/kg, twice daily) were administered. Seven days later, the portal vein thrombosis disappeared. Once daily oral rivaroxaban (10 mg) was resumed for 4 months without thrombosis (Figure 3).

### 3.5. Complications

No signs of portal hypertension and splenic abscess have been observed, and no other complications occurred.

## 4. Discussion

CEPS is a congenital malformation created by an abnormal shunt between the portal vein and vena cava or azygos/hemiazygos system due to abnormal development of the umbilical vein and yolk vein during the embryonic period. In combination with other congenital malformations, patients with CEPS present with hypergalactosemia, high bile acid, high serum ammonia, and hepatic encephalopathy caused by the portal and vena cava shunt. It has been reported that 66-100% of patients with CEPS have hyperammonemia and 17-30% have hepatic encephalopathy [5–7].

Currently, liver transplantation is the only curative treatment for type I CEPS. Surgical ligation and endovascular embolization have been recommended to treat symptomatic type II CEPS; however, no consensus has been achieved and no comparison between the two procedures has been reported. Except for patients who are not suitable for endovascular occlusion due to shunt anatomy, no research has compared the more advantageous modalities for patients suitable for both treatment procedures so far. Our study found that both procedures were safe and effective in patients with type II CEPS. However, compared with laparoscopic surgical ligation, the endovascular approach has the advantages of shorter procedure time, less intraoperative blood loss, and lower treatment expense.

Angiography is the gold standard for diagnosing CEPS and identifying the type of CEPS present [8, 9]. While shunt anatomy and flow dynamics can be determined by angiography and/or direct venography, balloon shunt occlusion venography is critical for detecting intrahepatic portal vein branches. This branch identification could also determine the type of CEPS and the pressure gradient pre- and postocclusion that is crucial for assessing the risk of portal hypertension after shunt occlusion [9–12].

In previous reports, 25 mmHg was used as a threshold to monitor PVP. If PVP was less than 25 mmHg after 15-20 min of occlusion, it indicated that the risk of portal hypertension was insignificant after shunt occlusion. Otherwise, a two-step closure is recommended with partial occlusion to initially acclimatize the intrahepatic portal system to increase flow and then completely occlude in 6 to 12 months [9, 13–16].

To avoid portal hypertension postocclusion for patients with PVP of more than 25 mmHg, we performed surgical ligation of the shunt combined with the splenic artery and/or vein and thereby avoided two surgical procedures for shunt closing, because the ligation of splenic artery and/or vein could reduce the PVP by reducing portal blood flow. Based on the abundant communication between the splenic artery, splenic vein, and splanchnic vessels, ligation of the splenic artery and splenic vein will not typically cause splenic necrosis or abscess. In this cohort, six patients underwent shunt and splenic artery ligation and four underwent additional splenic vein ligation. No portal hypertension or splenic abscess occurred, and a second surgical treatment was avoided. Therefore, surgical ligation could be used for these patients.

Portal vein and mesenteric venous thrombosis is a common complication after shunt ligation that can lead to portal hypertension; therefore, postprocedural anticoagulation therapy is very essential [4, 8]. Heparin and warfarin have been used to prevent thrombosis in previous studies; however, the activated partial thromboplastin time and international normalized ratio should be closely monitored to adjust drug dosage [14, 17]. In our practice, enoxaparin and rivaroxaban have been used sequentially for anticoagulation after occlusion and do not require monitoring indicators of coagulation and are more convenient to use. In the follow-up period, only one patient (1/23) suffered portal vein thrombosis, which disappeared after 7 days of conservative treatment.

Theoretically, the hypoplastic intrahepatic portal vein should dilate after the perfusion of portal vein blood increased due to shunt ligation. In this cohort, patients after endovascular embolization achieved a significant increase in portal vein diameter; however, there was no significant difference in surgical ligation. This may be attributed to the well-grown intrahepatic portal vein branches in several patients before occlusion and the limited number of cases. Additionally, during the follow-up, surgical candidates did a little worse than interventional group; this may be associated with these patients with worse-grade disease preprocedure.

Our study had some limitations. First, this study included only a small number of patients primarily due to the low incidence of CEPS. Second, the follow-up period was short, and due to the retrospective characteristics of the study, the follow-up intervals were not completely consistent and partial follow-up data was missing. Thirdly, the portosystemic gradient was not measured. Lastly, the patients of the two groups were divided according to the length and diameter of the shunt, so it might led to patient selection bias between the groups.

## 5. Conclusions

Both surgical ligation and endovascular embolization are effective in the treatment of type II CEPS. The endovascular approach has the advantages of shorter procedure time, less intraoperative blood loss, and lower treatment expense. Surgical ligation is recommended for patients with a high risk of embolic material migration due to short and large extrahepatic portosystemic shunts and patients with PVP more than 25 mmHg after occlusion, which could lead to portal hypertension. Ligation of the portosystemic shunt and the splenic artery and vein is feasible with apparent safety, and it could avoid a second surgical treatment.

## Figures and Tables

**Table 1:** Baseline characteristics of patients in both groups.

Characteristic	Surgical group (*n* = 13)	Interventional group (*n* = 10)	*P* value
Age	40.7 ± 20.4	44.9 ± 19.7	0.68
Male	5 (38.5)	8 (80.0)	
Female	8 (61.5	2 (20.0)	
CEPS symptoms			
Hepatic encephalopathy	6 (46.2)	5 (50.0)	>0.99
Gastrointestinal bleeding	4 (30.8)	2 (20.0)	0.66
Dyspnea	1 (7.7)	0 (0)	>0.99
Abdominal pain	1 (7.7)	1 (10.0)	>0.99
Hepatic myelopathy	1 (7.7)	1 (10.0)	>0.99
Hemoptysis	0 (0)	1 (10.0)	>0.99
Fatigue	2 (15.4)	1 (10.0)	>0.99
Comorbidity			
Hepatic cirrhosis	10 (76.9)	5 (50.0)	0.22
Hepatic adenoma	2 (15.4)	0 (0)	0.49
Pulmonary hypertension	1 (7.7)	1 (10.0)	>0.99
Hypersplenism	1 (7.7)	1 (10.0)	>0.99
Location of shunt vessels			
Splenorenal shunt	5 (38.5)	3 (30.0)	>0.99
SMV-IVC shunt	3 (23.1)	1 (10.0)	0.60
Portal vein-IVC shunt	3 (23.1)	2 (20.0)	>0.99
SMV-renal vein shunt	1 (7.7)	1 (10.0)	>0.99
Portal vein-renal vein shunt	1 (7.7)	1 (10.0)	>0.99
Portal vein-iliac vein shunt	0 (0)	2 (20.0)	0.18

Data are numbers of patients, with percentages in parentheses. SMV: superior mesenteric vein; IVC: inferior vena cava.

**Table 2:** Comparison of portal vein pressure in the surgical and interventional group pre- and postprocedure.

Variable	Surgical group (*n* = 13)	Interventional group (*n* = 10)	*P* value
Portal vein pressure (mmHg)			
Preprocedure	12.9 ± 3.7	15.9 ± 4.7	0.18
Postprocedure	17.5 ± 3.8	17.1 ± 4.8	0.86
*P* value	<0.001	0.22	

**Table 3:** Differences in intraoperative parameters between surgical and interventional groups.

Parameters	Surgical group (*n* = 13)	Interventional group (*n* = 10)	*P* value
Procedure time (min)	219.8 ± 56.7	127.0 ± 43.2	<0.001
Intraoperative blood loss (mL)	238.5 ± 396.9	32.0 ± 62.5	0.001
Treatment expense (yuan)	59561.3 ± 21450.1	46331.5 ± 18839.1	0.20

**Table 4:** Clinical outcomes in the surgical and interventional group pre- and postprocedure.

Variable	Surgical group (*n* = 13)	Interventional group (*n* = 10)	*P* value
Hemoglobin (g/L)			
Preprocedure	109.4 ± 26.2	102.0 ± 25.2	0.46
3 months	104.0 ± 27.0	112.4 ± 21.1	0.08
6 months	114.0 ± 8.5	125.0 ± 18.4	<0.001
12 months	104.3 ± 19.8	118.8 ± 18.9	<0.001
36 months	125.5 ± 24.7	127.7 ± 22.5	0.03
Serum ammonia (*μ*mol/L)			
Preprocedure	83.6 ± 32.5	75.1 ± 30.5	0.12
3 months	56.4 ± 20.8	47.8 ± 46.4	0.003
6 months	41.0 ± 1.4	51.4 ± 49.8	0.01
12 months	19.2 ± 6.8	13.5 ± 6.4	<0.001
36 months	24.0 ± 6.4	16.7 ± 6.2	<0.001
Portal vein diameter (mm)			
Preprocedure	8.5 ± 4.9	9.3 ± 4.0	0.25
3 months	9.6 ± 3.4	10.7 ± 2.7	0.34
6 months	9.7 ± 3.0	10.8 ± 2.6	0.22
12 months	10.0 ± 3.0	11.2 ± 2.9	0.09
36 months	10.2 ± 2.9	11.6 ± 2.3	0.046
Child-Pugh score			
Preprocedure	7.8 ± 1.7	7.3 ± 2.4	0.36
3 months	6.7 ± 1.9	5.8 ± 1.3	0.01
6 months	5.5 ± 0.7	6.0 ± 1.4	0.001
12 months	6.0 ± 1.4	5.2 ± 0.4	<0.001
36 months	5.5 ± 0.7	5.3 ± 0.6	0.001

## Data Availability

The data used to support the findings of this study are included within the article.
